# Overnight olfactory enrichment using an odorant diffuser improves memory and modifies the uncinate fasciculus in older adults

**DOI:** 10.3389/fnins.2023.1200448

**Published:** 2023-07-24

**Authors:** Cynthia C. Woo, Blake Miranda, Mithra Sathishkumar, Farideh Dehkordi-Vakil, Michael A. Yassa, Michael Leon

**Affiliations:** ^1^Department of Neurobiology and Behavior, University of California, Irvine, Irvine, CA, United States; ^2^Center for the Neurobiology of Learning and Memory, University of California, Irvine, Irvine, CA, United States; ^3^Department of Statistics, University of California, Irvine, Irvine, CA, United States; ^4^Institute for Memory Impairments and Neurological Disorders, University of California, Irvine, Irvine, CA, United States

**Keywords:** olfactory training, fMRI, environmental enrichment, uncinate fasciculus, cognitive loss, olfaction

## Abstract

**Objective:**

Cognitive loss in older adults is a growing issue in our society, and there is a need to develop inexpensive, simple, effective in-home treatments. This study was conducted to explore the use of olfactory enrichment at night to improve cognitive ability in healthy older adults.

**Methods:**

Male and female older adults (*N* = 43), age 60–85, were enrolled in the study and randomly assigned to an Olfactory Enriched or Control group. Individuals in the enriched group were exposed to 7 different odorants a week, one per night, for 2 h, using an odorant diffuser. Individuals in the control group had the same experience with *de minimis* amounts of odorant. Neuropsychological assessments and fMRI scans were administered at the beginning of the study and after 6 months.

**Results:**

A statistically significant 226% improvement was observed in the enriched group compared to the control group on the Rey Auditory Verbal Learning Test and improved functioning was observed in the left uncinate fasciculus, as assessed by mean diffusivity.

**Conclusion:**

Minimal olfactory enrichment administered at night produces improvements in both cognitive and neural functioning. Thus, olfactory enrichment may provide an effective and low-effort pathway to improved brain health.

## 1. Introduction

There is a clear need for a new approach to the treatment of cognitive loss in older adults that takes little effort but is highly effective and affordable (Swedish Council on Health Technology Assessments, 2008). Environmental enrichment has long been studied in rats and mice, which can be enriched by placing them in a large cage with conspecifics, a running wheel, and regularly changing physical elements, rather than their restrictive box cage (Kempermann, 2019). The enrichment stimulates neuroplasticity that improves their human-like neurological symptoms in more than two dozen animal models of human neurological disorders (Nithianantharajah and Hannan, 2009; Hannan, 2014; Kempermann, 2019). Environmental enrichment also has more specifically been shown to ameliorate the human-like cognitive decline in animal models of aging (Valero et al., 2011; Patel, 2012). In lab animals, enhanced visual (Iaccarino et al., 2016), auditory (Martorell et al., 2019; Jung et al., 2023), and mastication (de Siqueira Mendes et al., 2021) stimulation, facilitates memory as it does in human older adults (Leon and Woo, 2018; Chan et al., 2022).

### 1.1. Olfactory enrichment alone improves brain and behavior in laboratory animals

Olfactory enrichment involves the daily exposure of individuals to multiple odorants and Veyrac et al. (2009) showed that olfactory enrichment alone could improve both memory and neurogenesis in the mouse brain. They further showed that novelty was the critical element in this kind of stimulation, as exposure to odorant mixtures did not produce these changes, while exposure to multiple odorants individually did. Rusznák et al. (2018) also showed that exposure to various essential oils alone for 30 min/day over 3 months induced neurogenesis in both the olfactory bulb and the hippocampus.

The olfactory system is the only sensory system that has direct projections to the limbic system which is crucial for memory and emotion, and which is the most relevant for this investigation (Haberly and Price, 1977), while the other sensory systems have indirect connections to this region via the thalamus. This unique access to the brain’s learning and memory systems may allow the olfactory system to prevent or reverse the deterioration of these systems via direct neural activation.

### 1.2. Loss of olfactory ability and the deterioration of cognition

As people age, the deterioration of their olfactory ability occurs before the deterioration of their cognitive abilities (Doty et al., 1984; Schaie et al., 2004). Additionally, olfactory loss results in a significant loss of both gray matter and white matter in human brains (Bitter et al., 2010a,b; Segura et al., 2013; Kollndorfer et al., 2014; Yao et al., 2014). COVID-19 typically results in olfactory loss and can result in long-term cognitive loss (Meng et al., 2020; Graham et al., 2021). Moreover, comparisons of MRI scans from individuals both pre-infection and post-infection have revealed neural deterioration that resembles a decade of aging in brain regions that receive olfactory-system projections (Douaud et al., 2022). Even chronic sinusitis has been shown to be associated with a decrease in gray matter in brain regions associated with learning and memory (Han et al., 2017).

### 1.3. Olfactory loss precedes or accompanies cognitive decline in dementia

Olfactory loss predicts the loss of gray matter in the hippocampus of older adults and continuing loss of olfaction predicts the further loss of hippocampal gray matter as they first develop Mild Cognitive Impairment (MCI) and then Alzheimer’s disease (Franks et al., 2015; Chen et al., 2021). Degradation of olfactory ability predicts which individuals with MCI will develop Alzheimer’s disease (Conti et al., 2013). In addition, olfactory dysfunction predicts cognitive dysfunction in humans (Choi et al., 2018) and the loss of olfactory function precedes or parallels the onset of a wide variety of other conditions such as: Parkinson’s disease (Ponsen et al., 2004; Meusel et al., 2010), Lewy body dementia (Ross et al., 2006), frontotemporal dementia, semantic dementia, frontotemporal dementia, corticobasal degeneration (Luzzi et al., 2007), Creutzfeldt-Jakob disease (Tabaton et al., 2004), alcoholism (Rupp et al., 2003), and schizophrenia (Kopala and Clark, 1990; Nguyen et al., 2010). Douaud et al. (2022) found that the same areas that deteriorate in older adults or adults with olfactory loss was seen in people who had experienced a COVID infection, even an infection with mild symptoms.

### 1.4. Olfactory stimulation restores olfactory function

Olfactory enrichment improves olfactory ability in humans with olfactory loss due to post-infection olfactory dysfunction (Konstantinidis et al., 2013, 2016; Damm et al., 2014; Geißler et al., 2014), head trauma (Huang et al., 2021), Parkinson’s (Haehner et al., 2013), or aging (Zambom-Ferraresi et al., 2021). These results were achieved with daily exposure to four odorants that represented the resinous, flowery, fruity, and aromatic odor groups. There are further improvements in olfactory ability with increased duration of exposure (Altundag et al., 2015; Konstantinidis et al., 2016), increased concentration of the odorants (Damm et al., 2014), and an increased number of odorants (Mahmut et al., 2020).

### 1.5. Olfactory enrichment changes human brain anatomy

Al Aïn et al. (2019) found that olfactory enrichment improved odor identification compared to that of visually enriched controls. Moreover, MRI analysis showed that olfactory enrichment led to increased cortical thickness in the right inferior frontal gyrus, the bilateral fusiform gyrus and the entorhinal cortex when compared to controls. Gellrich et al. (2017) found that olfactory enrichment given to people with olfactory deficiencies increased gray matter volume in the hippocampus and the thalamus, but no the brain regions. Similarly, Han et al. (2021) gave older adults olfactory enrichment for 7 months using 4 odorants twice/day, and patients had improved odor identification skills and larger cortical gray matter volume relative to controls. Sommelier students are exposed to dozens of novel odorants each day of their training. Using a longitudinal design, the brains of sommelier students were imaged with MRI at the start and end of their 18-month training and were compared with control students (Filiz et al., 2022). Olfactory enrichment of sommelier students increased olfactory bulb volume and it also increased the thickness of the entorhinal cortex. There were no significant changes in control group brains.

### 1.6. Olfactory enrichment improves cognition in humans

Haehner et al. (2013) showed that patients with Parkinson’s disease improved their verbal fluency after olfactory enrichment. Birte-Antina et al. (2018) provided olfactory enrichment for adults with 4 essential-oil odorants twice a day for 5 months. Controls solved daily Sudoku puzzles during that time. The olfactory-enriched group had a significant improvement of olfactory function, improved verbal function, and decreased depression symptoms. Oleszkiewicz et al. (2021) exposed 68 older adults either to 9 odorants twice a day or to no new olfactory stimulation for 3–6 months, and found the enriched olfactory experience produced improvements in cognitive abilities, dementia status, and olfactory function, relative to the control condition. Specifically, the Montreal Cognitive Assessment revealed a significant difference between the olfactory-enriched group and controls. They also found that the AD8 Dementia Screening Interview showed that olfactory-enriched participants had no increase in dementia symptoms over the course of the trial, while control participants had such an increase. Finally, an improvement on olfactory sensitivity was seen in olfactory-enriched individuals, but not controls. At the same time, Chen et al. (2022) did not find memory improvement in older adults with mild cognitive impairment after brief exposures to multiple odorants twice each day for 4 months. They did find that olfactory-enriched individuals increased frontal lobe activation but had no change in gray matter volume. In a similar study, Haehner et al. (2022) found that improvements in olfactory discrimination, increased thickness of the hippocampus, and improved global cognition were associated with increased thickness of the hippocampus, entorhinal cortex, and medial temporal lobes. Moreover, the change in the thickness of entorhinal cortex was positively associated with improvement of executive function.

### 1.7. Increased complexity of olfactory enrichment remarkably improves dementia

Cha et al. (2022) exposed older adults with moderate dementia either to 40 odorants twice a day for 15 days or to no olfactory enrichment. The olfactory-enriched group showed highly significant improvements in memory, olfactory identification, depression symptoms, attention, verbal fluidity, and language skills relative to controls.

### 1.8. Olfactory enrichment at night

While sniffing 40 odorants twice a day benefits patients with dementia, it is unlikely that they would be able to load, open, and close 80 sniff bottles each day. This problem would be expected even in older adults without dementia. Since it is important to get high levels of compliance for olfactory enrichment to obtain maximal benefits, we tested the idea that we could get enhanced neural and cognitive outcomes after minimal-effort olfactory enrichment at night.

The goal of the study was to determine whether participants retain or improve their cognitive ability after olfactory enrichment at night. We tested our hypothesis that the cognitive benefits of olfactory exposure on cognition may be found in its privileged access to brain areas and pathways relevant to olfaction and memory where it may be normalizing specific memory circuits. Specifically, we used diffusion weighted imaging to assess whether major limbic pathways (i.e., the uncinate fasciculus and the cingulum) are modified by olfactory enrichment. We focused on the uncinate in particular as a major pathway connecting the basolateral amygdala and the entorhinal cortex to the prefrontal cortex (Ebeling and Von Cramon, 1992; Thiebaut de Schotten et al., 2012; Von der Heide et al., 2013) and which plays a crucial role in learning and memory (Alm et al., 2016) and which deteriorates with age and Alzheimer’s disease (Morikawa et al., 2010; Fan et al., 2019). Importantly, a recent study demonstrated that the uncinate fasciculus is modified by a dance intervention as a form of environmental enrichment (Rektorova et al., 2020). This motivated our choice of the uncinate fasciculus as a target region of interest to test for the effect of olfactory enrichment.

## 2. Materials and methods

### 2.1. Participants

**Figure 1:**
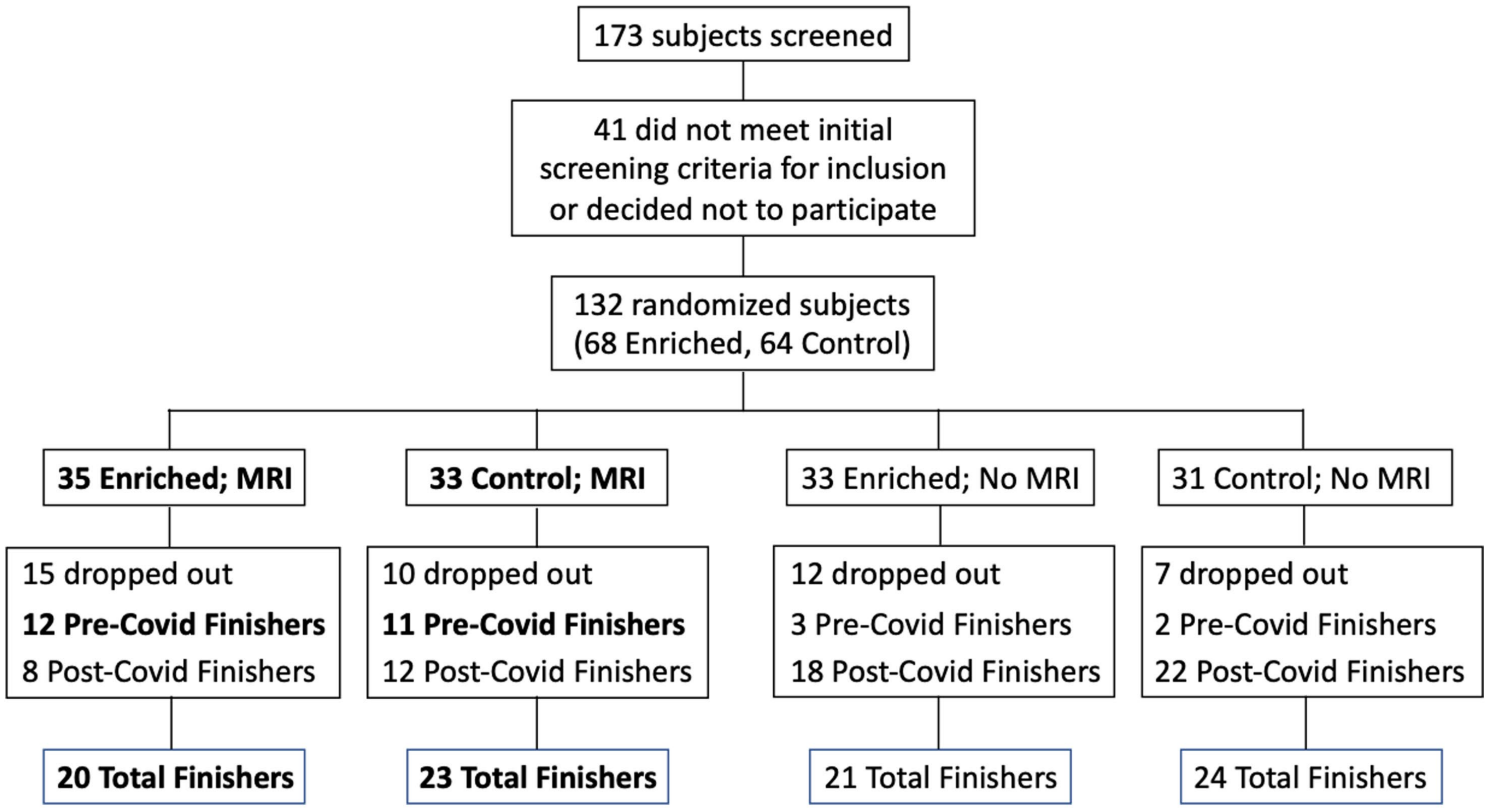
Flow chart for subject participation. Bold font denotes subgroups that were included in the statistical analyses (Pre-Covid Finishers, for neuropsychological assessments; and MRI Finishers, for MRI analyses).

Participants were recruited from a list of interested older adults via the UCI Institute for Memory Impairments and Neurological Disorders’ Consent-to-Contact Registry. The participants were all community-dwelling older adults with no diagnosis of cognitive impairment or dementia. They received monetary compensation for their participation. Informed consent was given by all participants, all procedures were approved by the UC Irvine Institutional Review Board, and we conformed to the principles of the Helsinki Declaration. All participants were screened against major medical or psychiatric morbidities (including head trauma), substance abuse history, and any MRI contraindications, such as metal in the body. Inclusion and exclusion criteria are shown in Table 1. This trial was registered at ClinicalTrials.gov (Identifier: NCT03914989). Participants were 43 male and female, age 60–85, of good general health, with normal cognition, was defined as greater than or equal to 24 on the MMSE (see Figure 1 for subject participation flowchart). Participant characteristics are shown in Table 2.

**Table 1:** Inclusion and exclusion criteria.

Inclusion criteria	Exclusion criteria
Age 60–85, male or female	Known odor sensitivities
Had normal cognition (determined at first assessment; MMSE ≥ 24)	Had asthma, allergies, or an odor response, which produces symptoms similar to those of an allergy, including runny nose, watery eyes, sneezing, or skin rash
Spoke/read/understood English fluently	Had a neurological disease such as Parkinson’s disease, multiple sclerosis, brain cyst, tumor, or aneurysm
Had visual and auditory acuity adequate for neuropsychological and computerized testing	Had major health conditions such as uncontrolled diabetes mellitus, uncontrolled hypertension, nutritional deficiency or thyroid disease
Were in good general health with no disease(s) expected to interfere with the study	Had significant psychiatric disorders such as schizophrenia, bipolar disorder, anxiety disorder, or attention-deficit hyperactivity disorder
Willing and able to participate for the duration of the study and in all study procedures	Had cognitive impairment when tested at baseline (defined as a score of <24 on baseline MMSE)
Were able to smell odors	Had had alcohol or substance abuse or dependence within the past 2 years (DSM-IV criteria)
Willing to refrain from using scented candles, scented oils, or air fresheners while participating in the study	Smoked cigarettes, e-cigarettes, cigars, marijuana, or any other substance that could produce an interfering odor for the study.
Willing to travel to the research site for testing	MRI contraindications, such as pacemakers, aneurysm clips, artificial heart valves, ear implants, metal fragments or foreign objects in the eyes, skin or body.

**Table 2:** Participant characteristics.

	Control	Enriched
Female	18	15
Male	5	5
Age: (Mean ± S.D.; range)	69.2 ± 7.1 years (62–85 years)	70.1 ± 6.6 years (60–81 years)
Education: (Mean ± S.D.; range)	16.0 ± 1.9 years (12–20 years)	16.1 ± 2.2 years (12–20 years)

We randomly assigned participants to receive daily exposure to essential oil scents (*N* = 20) or to a sham control condition with trace amounts of odorant (*N* = 23). CONSORT guidelines were adhered to in the analysis and reporting of study results. They were assessed for cognitive and olfactory abilities, as well as mental health status, at study entry and after 6 months. All participants received an odor diffuser to use at home for the 6-month duration of the intervention. The enriched group was exposed for 2 h every night over the course of 6 months, using a single odorant each night, and rotating through seven pleasant scents.

Participants provided Informed Consent, and completed a Background Information Questionnaire, a Fragrance Usage Survey, and a Daily Activities Questionnaire. The Background Information Questionnaire included age, education level, daily activities, medication, and emergency contact information. The Fragrance Usage Survey provided information regarding their typical daily odorant usage. The Daily Activities Questionnaire included the activities in which they participated that week. We used the Mini-Mental State Examination (MMSE) to confirm normal cognitive functioning.

### 2.2. Assessments and evaluations

We conducted a short neuropsychological test battery at baseline and after 6-month follow-up in all participants. To assess verbal learning and memory we used the Rey Auditory Verbal Learning Test (Rey, 1941), which assesses learning over five trials, followed by immediate and delayed recall tests. This test is also sensitive to both the integrity of the hippocampus and to the early detection of cognitive dysfunction (Saury and Emanuelson, 2017). Participants additionally completed three subsets of the Weschler Adult Intelligence Scale – Third edition (WAIS-III): the Digit Span Test (working memory) forward and backwards, and the Letter-Number Sequence test (planning and attention switching). We used Sniffin’ Sticks (Sensonics) to assess olfactory system function (olfactory identification, discrimination, and threshold) at baseline, allowing us to screen for olfactory abnormalities as well as to determine if olfactory enrichment enhanced olfactory performance.

### 2.3. Study intervention

Individuals assigned to the olfactory enrichment group were provided with an odorant diffuser (Diffuser World) and 7 essential oil odorants (rose, orange, eucalyptus, lemon, peppermint, rosemary, and lavender; from The Essential Oil Company, Portland, OR) in identical glass vials that each fit into the diffuser. They were asked to turn on the diffuser when they went to bed, and the odorant was released into the air during the night for 2 h when they first went to sleep. They rotated through the different odorants each night. Individuals in the control group also were provided with an odorant diffuser, and they followed the same regimen as the olfactory enrichment participants, however they were provided with bottles that contained distilled water with an undetectable, *de minimis* amount of odorant added. Participants were instructed to change the odorant bottle daily before they went to bed, and they continued this regimen at home for 6 months. Odorant bottles for both groups were labeled with the odorant name, and they were weighed prior to distribution, to obtain a baseline weight for the filled bottles, and then weighed again after 6 months to be sure that they were in use during the study. During each participant’s first visit, they smelled each of the scents used in the study and rated them on pleasantness and intensity.

We remained in contact with the participants during the first few days of the intervention to ensure adherence. We contacted each participant once a month during the intervention period to check on adherence, troubleshoot any issues, and inquire about any changes in health, or major life events. In addition, participants were asked to complete a daily Sensory Enrichment Log, which involved tracking the odorant exposures as they were completed, the time they went to sleep, and the approximate number of hours they slept that night.

### 2.4. Imaging methods

#### 2.4.1. Acquisition

All MRI data were collected using a 3.0 Tesla Siemens Prisma scanner with a 32-channel head coil at Facility for Brain Imaging Research at UC Irvine. A high-resolution three-dimensional (3D) rapid-gradient echo (MP-RAGE) structural scan was acquired (0.8 mm^3^ isotropic, TR/TE = 2300/2.38 ms, 240 slices, FOV = 256 × 256, flip angle = 8^o^, slice orientation = sagittal, GRAPPA acceleration factor = 3). Diffusion data were acquired in two b-shells: 1500 s/mm^2^ and 3000 s/mm^2^, 64 non-collinear directions and a single volume with a *b*-value of 0 s/mm^2^. (TR/TE = 3500/102 ms, FoV = 218 mm, slices = 72, voxel size = 1.7 × 1.7 × 1.7 mm, Interleaved slices, Slice acceleration factor = 4).

#### 2.4.2. Processing and analysis

DTI data collection and analysis were the same as for Granger et al. (2021). Motion correction and Eddy current correction were applied to raw data using FSL’s eddy tool (Andersson and Sotiropoulos, 2016). Corrected data were reconstructed using Q-spin Diffeomorphic Reconstruction (QSDR) function (Yeh and Tseng, 2011) in DSI Studio^1^, which uses a diffeomorphic algorithm to warp model-free orientation functions (ODFs) to Montreal Neurological Institute (MNI) space template. ODFs were reconstructed with the default diffusion sampling 1.25, which allowed modeling of crossing fibers at the intersection of the corticospinal tract and corpus callosum. Other reconstruction parameters included the following registration method: norm 7-9-7, eightfold ODF tessellation, number of fibers resolved. Output resolution was increased to 1 mm. Subject head motion was assessed by the eddy movement rms file exported from *eddy* (movement relative to the previous volume) and included as a nuisance regressor. T1-weighted MPRAGE scans were used to obtain intracranial brain volume using Freesurfer 6.0. The Johns Hopkins white matter atlas (in MNI space) in DSI studio contains various masks of regions of interest (ROIs) including limbic white matter regions, the uncinate fasciculus (UF) and the hippocampal cingulum bilaterally. We chose these two white matter pathways as major limbic-to-prefrontal tracts that are crucial to learning and memory.

Mean Diffusivity (MD), the average water diffusion rate within brain tissue, was extracted for each ROI. It was calculated as the mean of the three eigenvalues of the diffusion tensor vector (Salat, 2014). Generalized Fractional Anisotropy (GFA) values were extracted for each ROI and averaged across voxels. They were then averaged across both hemispheres. GFA is a model-free diffusion measure and is known to correlate with fractional anisotropy (FA) of the tensor model. GFA has been previously used to assess the structural integrity of complex tissues in a clinical setting, particularly when there are heterogeneous fiber tissues (Koh et al., 2018).

### 2.5. Impact of COVID-19 pandemic

Due to the COVID-19 pandemic, the UCI campus was closed in April 2020, and remained closed until the Fall of 2020. In addition, many participants did not feel comfortable entering the campus due to COVID-19 concerns even after the campus was officially open. As a result, participants who would have completed their 6-months of participation after April 2020 were either not able to return or chose not to return to campus for their second assessment. During the campus shutdown, contact was maintained with the participants who were impacted, and they were encouraged to continue their sensory enrichment, however, compliance was variable. When it became clear that the campus was going to remain closed for an extended period, we developed methods to remotely conduct the cognitive assessments using videoconferencing (Zoom app). When the campus re-opened and research participants were allowed back onto campus, participants who had received MRI scans at baseline received their second MRI scan.

The data set used for the cognitive assessment analysis was reduced due to a number of possible confounding issues including the different conditions present for the cognitive assessment testing at baseline (in office) and that given remotely (in their home using videoconferencing), the possible sensitivity of that testing to the immediate physical environment during the assessment, as well as the variable timing both between the date of the baseline assessment and the date of final assessment, and the date of their final assessment and the date they discontinued their sensory enrichment. Accordingly, in our data analysis for cognitive assessment, we only included individuals who had completed their 6-months of participation prior to the UCI shutdown (a total of 11 controls and 12 enriched). For the MRI analysis, we included everyone who returned to campus for their follow-up MRI despite the difference in time (range: 6–17 months; a total of 23 controls and 20 enriched).

### 2.6. Statistical analysis

The key analysis was a 2 × 2 mixed ANOVA with Time (Pre vs. Post) as the repeated (within subjects) measure and Group (Control vs. Treated) as the across-subjects measure. Given the matching of other variables including age, gender and years of education, we did not include these variables as covariates in the model. A two-sided P less than 0.05 was considered statistically significant. We used SAS9.4 for all statistical analyses.

## 3. Results

**Figure 2:**
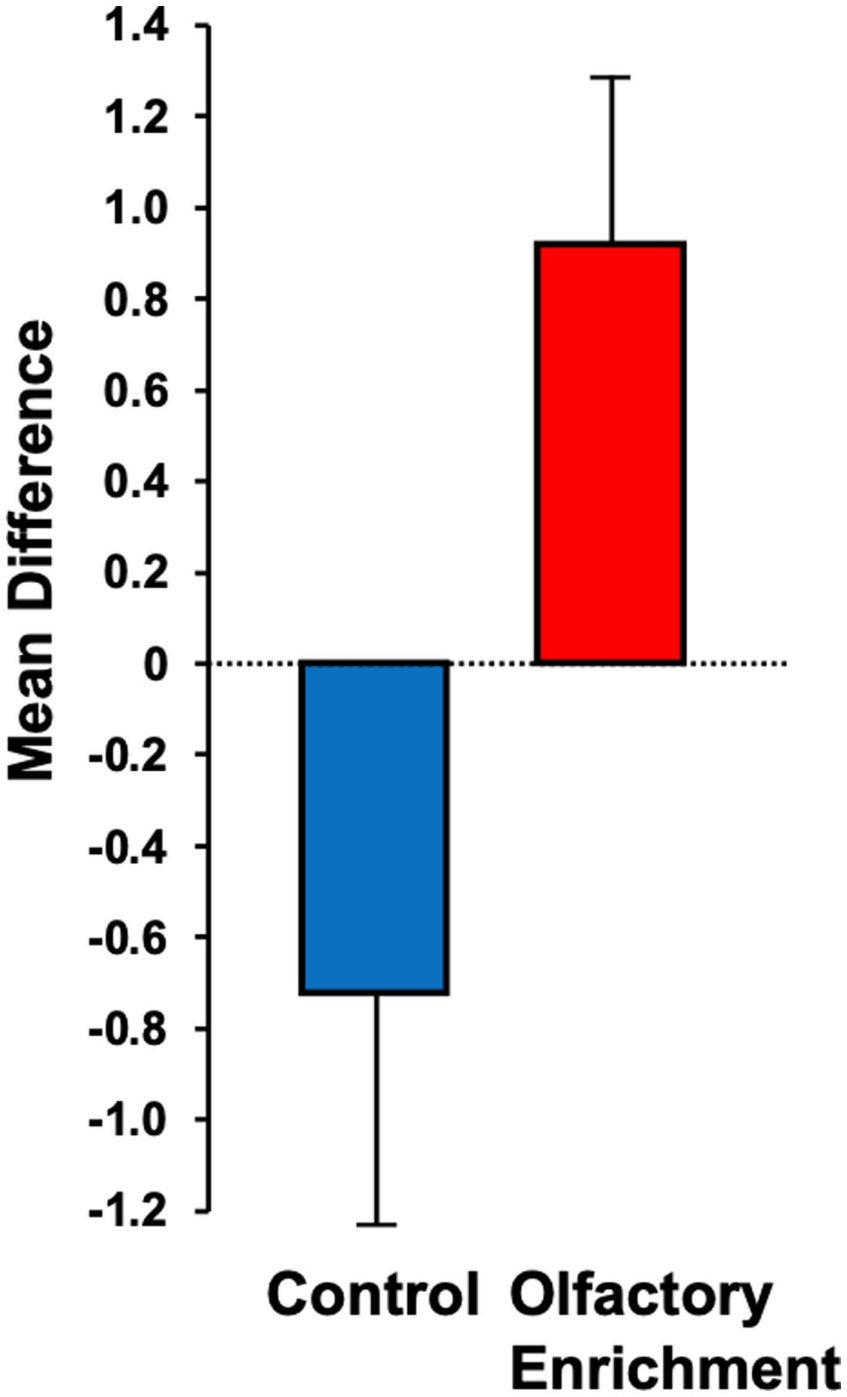
Mean difference between pre- and post-measurements for the Rey Auditory Verbal Learning Test (RAVLT – A5). Statistically significant difference between groups using an ANOVA with repeated measures (*p* = 0.02).

Although we used 7 odors in total with 2 h of olfactory stimulation each night, we only exposed participants to one odor each night, while others have used a minimum of four odors each day for olfactory enrichment (Pieniak et al., 2022). Despite the minimal variety of olfactory exposure each night, we observed a clear, statistically significant (Timepoint x Group interaction, *F* = 6.63, *p* = 0.02, Cohen’s *d* = 1.08, a large-size effect) 226% difference between enriched and control older adults in performance on the Rey Auditory Verbal Learning Test (RAVLT; last learning trial A5; Figure 2). This test evaluates verbal learning and memory, including proactive interference, retroactive interference, delayed recall, retention, and recognition memory. We found that 3 of 11 Controls improved, 1 of 11 stayed the same, 7 of 11 did worse. Among the Enriched group, 6 of 12 improved, 5 of 12 stayed the same, 1 of 12 did worse. Improvements with enrichment continued for retention trials (A6 and A7) following the interference list (B1), although those differences were just shy of statistical significance (*F* = 3.98, *p* = 0.06 for A6 and *F* = 3.69, *p* = 0.07 for A7). No other differences were observed in the other assessments (Table 3).

**Table 3:** Cognitive assessment results.

Assessment	Mean difference between pre- and post- measurements (Mean ± S.D.)	ANOVA repeated measures (Group × Measurement Time) *F*-value	ANOVA repeated measures (Group × Measurement Time) *P*-value
MMSE
Control	−0.36 ± 2.34	0.02	0.89
Enriched	−0.50 ± 2.24		
RAVLT A1
Control	0.36 ± 1.91	0.07	0.79
Enriched	0.17 ± 1.64		
RAVLT A2
Control	−0.36 ± 2.29	0.10	0.76
Enriched	−0.67 ± 2.35		
RAVLT A3
Control	0.27 ± 2.61	0.12	0.73
Enriched	0.58 ± 1.56		
RAVLT A4
Control	−0.45 ± 2.38	1.63	0.22
Enriched	0.58 ± 1.44		
RAVLT A5
Control	−0.73 ± 1.74	6.63	0.02*
Enriched	0.92 ± 1.31		
RAVLT B1
Control	0.09 ± 2.88	0.10	0.76
Enriched	−0.25 ± 2.42		
RAVLT A6
Control	−0.36 ± 2.06	3.98	0.06
Enriched	1.25 ± 1.82		
RAVLT A7
Control	−0.55 ± 2.16	3.69	0.07
Enriched	1.33 ± 2.50		
WAIS digit span forward
Control	0.00 ± 1.79	0.06	0.80
Enriched	−0.17 ± 1.34		
WAIS digit span backward
Control	−0.45 ± 2.88	0.41	0.53
Enriched	0.17 ± 1.64		
WAIS letter/number
Control	0.55 ± 2.73	0.26	0.61
Enriched	1.08 ± 2.31		

**Figure 3:**
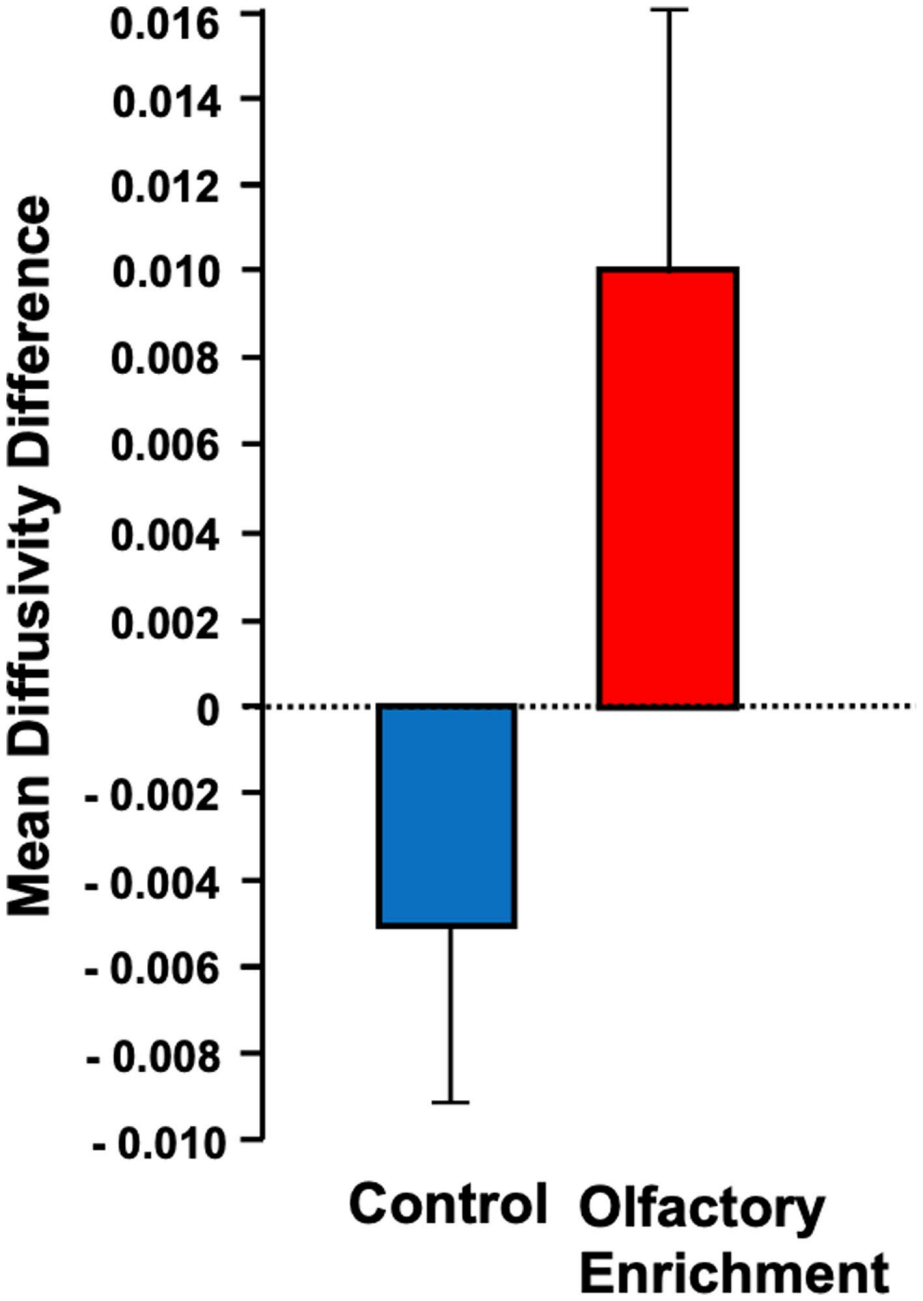
Shows the mean diffusivity difference between olfactory-enriched and control older adults. Statistically significant difference between groups using an ANOVA with repeated measures (*p* = 0.04). Error bars denote standard error of the mean (SEM).

We also found a significant change (Timepoint x Group interaction, *F* = 4.39, *p* = 0.043, *η*^2^*p* = 0.101, a medium-size effect) in the mean diffusivity (MD) of the left uncinate fasciculus in the enriched group compared to controls (Figure 3). We found no other significant differences in these measures. The degree of change in the brain measure was not significantly correlated with the degree of change in the behavioral measure (*p* > 0.05) but this may be due to the reduced power in this analysis which necessarily only included the smaller subset of individuals who completed neuropsychological assessments during in-person visits.

For the RAVLT, Control females (*N* = 9) decreased their scores by 0.1 points and Control males (*N* = 2) decreased their scores by 3.5 points, while Enriched females (*N* = 8) increased their scores by 0.88 points and Enriched males (*N* = 4) increased their scores by 1 point. Controls between the ages of 60–72 years old (*N* = 10) decreased their scores by 0.7 points, while Enriched participants in that age range (*N* = 7) improved by 1.29 points. Control participants who were between 73 and 85 years old (*N* = 1) decreased by 1.0 point, while Enriched participants in that age range (*N* = 5) improved by 0.4 points.

For the MD differences in the uncinate fasciculus, Control females (*N* = 18) decreased by 0.008, while Control males (*N* = 5) increased by 0.004. Enriched females (*N* = 15) increased by 0.01, while Enriched males (N-5) increased by 0.007. Controls 60–72 years old (*N* = 17) decreased by 0.009, while participants who were 73–85 years old (*N* = 6) increased by 0.007. Enriched participants 60–72 years old (*N* = 13) increased by 0.003, while those between 73–85 (*N* = 7) increased by 0.02.

To examine any changes in sleep duration, we calculated the difference between the average sleep duration for the first 14 days (baseline) and that for the last 14 days (6-months), and compared differences across the two groups. We found a slight increase in the average amount of sleep recorded by subjects in the Enriched group (22 min) compared to that in the Control group (−3 min), however the difference did not reach statistical significance (*p* > 0.05). We found no statistically significant differences between groups on their olfactory ability, including threshold, discrimination, and recognition.

## 4. Discussion

The goal of the study was to determine whether older adults retain or improve their cognitive ability over a six-month period after daily olfactory enrichment at night. We found that, compared to controls, enriched participants improved in their performance on word list recall, a key test of verbal learning and memory. Cha et al. (2022), also found significant improvements in memory using a word list recall test similar to the RAVLT after olfactory enrichment in older adults with dementia. Birte-Antina et al. (2018) gave olfactory training to older adults and found significant improvements in the semantic-categorical verbal fluency portion of the Controlled Oral Word Association Test (COWAT) and the short-term memory section of the Montreal Cognitive Assessment test (MoCA). Oleszkiewicz et al. (2021) found that the control group had cognitive decline while the olfactory training groups did not. Oleszkiewicz et al. (2022) found that verbal fluency significantly improved for an odor-exposure group.

Additionally, we found that the mean diffusivity in the uncinate fasciculus increased in response to olfactory enrichment. The uncinate fasciculus is a major pathway that connects the basolateral amygdala and the entorhinal cortex to the prefrontal cortex (Ebeling and Von Cramon, 1992; Thiebaut de Schotten et al., 2012; Von der Heide et al., 2013). This brain pathway deteriorates in aging and furthermore in Alzheimer’s disease (Morikawa et al., 2010; Fan et al., 2019) and has been suggested to play a role in mediating episodic memory, language, socio-emotional processing and selecting among competing memories during retrieval (Alm et al., 2016).

Changes in mean diffusivity of white matter pathways have been previously observed with age. For example, white matter diffusivity decreases as adults age (Sexton et al., 2014). In general, mean diffusivity increases have been reported with enrichment interventions. For example, mean diffusivity of white matter in older adults increased when dancing was used as a form of environmental enrichment (Rektorova et al., 2020). Sensory enrichment provided by listening to music increased quantitative anisotropy longitudinally in the left uncinate fasciculus compared to the group that listened to audiobooks (Sihvonen et al., 2022). Widespread increases in mean diffusivity in the white matter tracts in the frontal lobes bilaterally, internal and external capsules, and partial right parietal lobe also occurred after a wellness/cognitive enhancement program in older adults (Stephen et al., 2020). Environmental enrichment in mice resulted in increased mean diffusivity in the visual cortex (Manno et al., 2022). Despite these results, at least one report has shown that mean diffusivity in the uncinate decreases with exercise (Predovan et al., 2021), however, it is important to note that the approach used in this study used probabilistic tractography of the uncinate and not an anatomical approach that consider the unique anatomy of this pathway [the approach we used in this study, and which is based on our prior work (Granger et al., 2021)]. This could explain the differences in the findings reported.

Olfactory stimulation does not go through the thalamus (Courtiol and Wilson, 2015; Gaeta and Wilson, 2022), a brain area that connects to the sleep control areas of the brain (Velluti, 2003; Carskadon and Herz, 2004), thereby preventing the odors from provoking a conscious perception during sleep. At the same time, olfactory stimulation during sleep deepens slow-wave sleep (Wolfe and Herzberg, 1996; Goel et al., 2005), which is the most restful portion of the sleep cycle, and people report feeling more vigorous the next day after nighttime olfactory exposure (Goel et al., 2005). Odorants enhance normal sleep, and they also improve abnormal sleep at a magnitude similar to that of sleep medication (Hardy and Kirk-Smith, 1995).

We have shown that even minimal olfactory enrichment, delivered at night, is sufficient to induce an improvement in cognition and neural function. This type of sensory enrichment may be particularly useful, as it is low cost, as well as low effort. This type of enrichment also appears even to be capable of successfully improving the cognitive ability of individuals living with dementia (Cha et al., 2022).

Limitations of the study include its small sample size and the single odorant that could be diffused each night due to the design of the diffusion device.

How might olfactory loss and olfactory enrichment impact cognition? It is striking how many human neurological disorders are accompanied by olfactory loss. Indeed, we have counted about 70 neurological and psychiatric disorders that are accompanied by olfactory loss (Doty and Hawkes, 2019; Leon and Woo, 2022). The wide range of etiologies and symptoms that encompass these cognitive, emotional, and motor problems may exist due to a common dysfunction, but they could reveal a fundamental role that olfactory impairment has in these disorders. Lifetime olfactory stimulation may have a salutary effect on the brain that may be similar to the concept of a cognitive reserve, wherein people who have had high levels of cognitive stimulation in life are protected from the neuropathology of Alzheimer’s dementia (Pettigrew and Soldan, 2019; Zijlmans et al., 2022). Conversely, the illiterate are 2–3 times as likely to have Alzheimer’s (Arce Rentería et al., 2019). Therefore, different levels of intellectual stimulation seem to increase or decrease the likelihood of developing dementia. In fact, there is a positive association between olfactory function and a cognitive reserve index (Masala et al., 2022).

It is possible that high levels of olfactory stimulation are protective for the brain and that the symptoms of these neurological disorders only become evident when olfactory stimulation is low. Perhaps this overarching influence of olfactory stimulation on neurological function underlies the remarkable finding in multiple large prospective studies that olfactory ability predicts all-cause mortality in adults (Gopinath et al., 2012; Liu et al., 2019; Choi et al., 2021; Pang et al., 2022). It therefore may be appropriate to begin envisioning olfactory enrichment as a low-cost public health program to reduce neurological risk in older adults.

We have shown that minimal olfactory enrichment at night using an odorant diffuser results in significant improvements in both verbal memory and the integrity of a specific brain pathway. Our findings should stimulate larger scale clinical trials systematically testing the therapeutic efficacy of olfactory enrichment in treating memory loss in older adults.

## Data availability statement

The datasets presented in this article are not readily available because due to reasonable privacy and security concerns, the underlying data are not easily redistributable to researchers other than those engaged in the current project’s Institutional Review Board-approved research. The corresponding author may be contacted for an IRB-approved collaboration. Requests to access the datasets should be directed to CW, cwoo@uci.edu.

## Ethics statement

The studies involving human participants were reviewed and approved by UC Irvine Institutional Review Board. The patients/participants provided their written informed consent to participate in this study.

## Author contributions

CW, MS, ML, and MY: study design, data interpretation, and manuscript writing. ML wrote first draft of manuscript. CW, BM, and MS: subject enrollment and follow-up, execution of study, and data analyses. FD-V: statistical analyses. All authors contributed to the article and approved the submitted version.

## Funding

This work was supported by Procter and Gamble.

## Conflict of interest

ML and MY have received travel expenses and compensation following presentations at P&G.

The remaining authors declare that the research was conducted in the absence of any commercial or financial relationships that could be construed as a potential conflict of interest.

## Publisher’s note

All claims expressed in this article are solely those of the authors and do not necessarily represent those of their affiliated organizations, or those of the publisher, the editors and the reviewers. Any product that may be evaluated in this article, or claim that may be made by its manufacturer, is not guaranteed or endorsed by the publisher.

## Footnotes

^1^ https://dsi-studio.labsolver.org/
